# Assessment of Pharmaceutical Company and Device Manufacturer Payments to Gastroenterologists and Their Participation in Clinical Practice Guideline Panels

**DOI:** 10.1001/jamanetworkopen.2018.6343

**Published:** 2018-12-28

**Authors:** Salman Nusrat, Taseen Syed, Sanober Nusrat, Sixia Chen, Wei-Jen Chen, Klaus Bielefeldt

**Affiliations:** 1 Section of Digestive Diseases and Nutrition, Department of Medicine, University of Oklahoma Health Sciences Center, Oklahoma City; 2 Section of Digestive Diseases and Nutrition, Department of Medicine, Veterans Affairs Medical Center, Oklahoma City, Oklahoma; 3 Department of Biostatistics and Epidemiology, University of Oklahoma Health Science Center, Oklahoma City; 4 Section of Gastroenterology, Department of Medicine, George E. Wahlen Veterans Affairs Medical Center, Salt Lake City, Utah

## Abstract

**Importance:**

Payments from pharmaceutical and device manufacturers to physicians may influence the advice physicians give patients and peers.

**Objectives:**

To investigate the nature and amounts of monetary and other benefits that gastroenterologists received and to determine the participation of those receiving benefits in the formulation of clinical practice guidelines.

**Design, Setting, and Participants:**

This cohort study analyzed information from the Centers for Medicare & Medicaid Services Open Payments database, including all reports about payments that pharmaceutical and device manufacturers gave to adult or pediatric gastroenterologists in 2016. PubMed was used to examine the professional affiliations and publication records of top payment recipients. Panelists of clinical guidelines who also received personal financial rewards listed in the Open Payments database were identified.

**Main Outcomes and Measures:**

Payments made to gastroenterologists by pharmaceutical company and device manufacturers.

**Results:**

Of 15 497 gastroenterologists, 13 467 (86.9%) received a total of 432 463 payments accounting for a total expenditure of $67 144 862. Direct financial payments for consultations, talks, or other services were made to 2055 physicians and were responsible for 4.2% of payments (18 179 of 432 463), but for 62.7% of total expenditures ($42 086 207 of $67 144 862). Although a significant number of submissions were for food and beverages, they constituted only a small amount of total expenditure. For gastroenterologists treating adult patients, 10 products were linked to 63.8% of payments (11 221 of 17 588) related to direct financial rewards and 37.1% of the total expenditures ($24 892 643 of $67 144 862). Twenty-nine of 36 clinical practice guidelines included panelists who had received honoraria or consultation fees from industry sources, with amounts exceeding $10 000 in 8 of them (22%).

**Conclusions and Relevance:**

Most gastroenterologists accept meals or gifts from industry, with 2055 of 15 497 gastroenterologists receiving direct payments and 8 of 36 clinical practice guidelines panelists having received more than $10 000. Considering the known impact of such benefits on prescribing patterns and other professional behaviors, policy makers should consider revising regulations governing interactions with industry and disclosure formats alerting others to their potential biasing impact.

## Key Points

**Question:**

How common are payments from pharmaceutical companies and device manufacturers to gastroenterologists?

**Finding:**

This cohort study of 15 497 gastroenterologists found that 86.9% received industry payments, with direct financial awards accounting for 62.7% of the total expenditures and 10 drugs accounting for 63.8% of the total payments. Twenty-nine of 36 recent guidelines included authors who had received industry payments.

**Meaning:**

While mandated reporting of industry payments to physicians provides transparency, the practice remains common and may continue to affect the advice these physicians give to patients and peers.

## Teaser

This cohort study investigates industry payments to gastroenterologists and evaluates participation by compensated physicians in the formulation of clinical practice guidelines.

## Introduction

Collaboration between health care professionals and the pharmaceutical industry often drives medical progress and, thus, benefits patients. Clinical trials reflect this reality, as industry sources support about half of the investigations focused on drugs or devices, raising concerns about potential conflicts of interest (COIs).^1^ Systematic reviews suggest an apparent bias of published studies that were more likely to report positive findings when the authors received support from industry.^2,3^ While journal editors responded to these findings by mandating disclosures of potential COIs,^4,5,6^ the possible extent of industry sponsorship came to light in the course of investigations by the Department of Justice, which led to a settlement between the United States and 5 companies owing to concerns about violations of federal anti-kickback regulations, as physicians had received consulting fees for using joint implants produced by these companies.^7^

Partly driven by this settlement, the Affordable Care Act of 2010 included a section that mandates reporting of all payments made by the pharmaceutical companies and device makers to health care professionals and teaching hospitals: The Physician Payment Sunshine Act. All transactions are now publicly recorded under the National Physician Payment Transparency Program of the Centers for Medicare & Medicaid Services (CMS).^8^ This Open Payments database of CMS facilitates the identification and tracking of COIs.^9,10^ However, it is unclear whether it changed behaviors, as rewards remain common and often involve physicians who play a role in formulating treatment guidelines that affect products of companies that supported them.^11,12^

Between 2014 and 2016, expenditures for pharmaceutical products outpaced inflation, increasing by a total of 20.9% in the United States and accounting for 46% of all sales recorded worldwide.^13^ This dramatic increase is not only driven by pricing strategies, but also represents a shift to designer drugs, such as antiviral drugs or cell cycle checkpoint inhibitors, that target smaller markets and often come with significantly higher costs. Considering the rapid growth of drug-related expenses, we decided to investigate payments and benefits received by gastroenterologists (GIs), specifically focusing on direct personal financial rewards. The underlying hypothesis was that high-priced specialty products may have disproportionate expenditures for rewards targeting physicians to more effectively penetrate a relatively small niche market. Therefore, we also looked at clinical practice guidelines published in a related 2-year window to determine industry payments to panelists involved in the formulation of such guidelines.

## Methods

We accessed the Open Payments database^14^ of CMS and extracted all reported transactions for the year 2016. We restricted the analysis to physicians listed as gastroenterologists, pediatric gastroenterologists, hepatologists, and transplant hepatologists. As submissions did not consistently use the various subspecializations of internal medicine, we bundled results for gastroenterologists, hepatologists, and transplant hepatologists as adult GIs, and contrasted them with submissions related to pediatric GIs. Individuals were identified based on the unique profile identification number assigned by CMS. We extracted name, city and state of practice, company submitting the payment report, nature of the payment, relationship of the payment to a specific product, name of the primary product linked to the transaction, paying company, and amount or value of the benefit received. The database categorizes the nature of benefits or payments as charitable giving; compensation for services other than consulting, including serving as faculty or as a speaker at a venue other than a continuing education program; compensation for serving as faculty or as a speaker for a nonaccredited and noncertified continuing education program; consultation fee; education; food and beverages; honoraria; royalty or license; travel or lodging; and current or prospective ownership or investment interest. Considering conceptual overlap, we bundled compensation and honoraria under a single category of compensation. Reports in these categories did not provide additional details about services or activities linked to the payment. There was only 1 report for charitable giving, which was not included in the analysis.

We first calculated total expenditures and number of recipients for the different categories, separating by specialties. We focused on personal financial rewards and summarized the total amount of money received for each individual captured, and then specifically determined amounts listed as compensation or consultation fees. We also recorded the number of companies and products associated with these payments. We sorted the top recipients in adult GI and pediatric GI, performed literature searches using the PubMed search engine, and counted publications for which these persons were authors for the years 2016 and 2017. We also assessed their affiliation with academic institutions using authorship information submitted to PubMed or practice location data based on name, city, and state as listed in Open Payments.

We accessed the websites of the American Board of Pediatrics^15^ and the Association of American Medical Colleges^16^ to obtain the number of physicians certified in the fields of pediatric and adult GI, respectively. For the 10 agents linked to most financial rewards, we determined the approval date listed on the website of the US Food and Drug Administration^17^ for the relevant indication (Table 1). In addition, we determined the annual sales in the United States and their relative weight in the context of the annual revenue by reviewing investor statements published by the companies.

**Table 1.** Consultation Fees Paid for the 10 Agents With the Highest Number of Direct Financial Rewards

Agent	Approval Date^a^	Consultations, No.	Recipients, No.	Compensation Amount, $	% of Total Payments	Average Retail Cost of Drug, $
Humira (adalimumab)	2012	189	113	491 442	22.9	5532 for 2 pens of Humira 40 mg/0.8 mL
Daklinza (daclatasvir)	2015	331	142	459 466 7	58.4	21 932 for 28 tablets
Viekira (ombitasvir, paritaprevir, and ritonavir)	2016	164	76	452 223	21.6	29 004 for 112 tablets
Epclusa (sofosbuvir and velpatasvir)	2016	131	98	389 440	12.0	20 376 for 28 tablets
Harvoni (ledipasvir and sofosbuvir)	2014	333	217	369 170	32.7	94 500 for 12-wk treatment regimen
Remicade (infliximab)	1998	114	41	278 032	27.5	750-915 for 1 vial of 100-mg strength
Stelara (ustekinumab)	2016	110	53	276 083	16.5	10 770 for 0.5 mL
Linzess (linaclotide)	2012	68	22	164 422	1.6	413 for thirty 72-μg capsules
Entyvio (vedolizumab)	2014	118	90	134 859	20.3	5782 per vial
Xifaxan (rifaximin)	2015	21	20	49 600	2.8	2586 for sixty 550-mg tablets

^a^ Approval dates are for the most recent change in indication, as listed by the US Food and Drug Administration.

Finally, we reviewed all guidelines published in 2016 and 2017 by the American Gastroenterological Association,^18,19,20,21,22,23,24,25,26,27,28,29,30,31,32,33,34,35^ the American College of Gastroenterology,^20,36,37,38,39,40,41,42,43,44,45,46,47,48,49^ the American Association for the Study of Liver Disease,^50,51^ and the North American Society for Pediatric Gastroenterology, Hepatology and Nutrition.^52,53,54,55,56^ We extracted names of participating panelists practicing in the United States based on their cited affiliations and reviewed the COI statements. Correcting for potential involvement in several guideline panels, we linked the names with names in the Open Payments database, ensuring complete concordance of first and last names and city of practice. We extracted the total sum paid as compensation or consulting fees by industry sources to these individuals and abstracted products the report listed as related to these rewards. As the CMS records capture payments to physicians licensed and practicing in the United States, we restricted our analysis to these persons.

We performed statistical analyses using Stata statistical software version 14 (StataCorp). Our primary outcome variable was the direct financial gain physicians received from the pharmaceutical industry. We show summary data of continuous variables as medians with a 95% confidence interval given in parentheses. For group comparisons, we considered a 2-sided *P* value of .05 to be statistically significant. We followed Strengthening the Reporting of Observational Studies in Epidemiology (STROBE) reporting guideline for cohort studies for our study design. Per institutional policy, institutional review board approval was not required.

## Results

**Figure 1.**
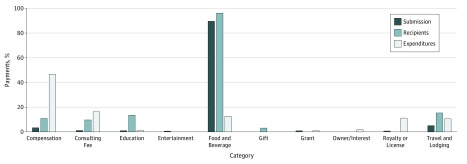
Relative Distribution of Payments by Nature of Rewards Data were normalized by the total number of reports (dark blue bars), the total number of recipients listed (light blue bars), and the total expenditures for the year 2016 (white bars).

We identified 15 497 practicing GIs and 13 467 of them (86.9%) were listed as adult or pediatric GIs, accounting for a total of 432 463 payments. When we separated data by professional groups, there were 12 553 adult GIs (93.2%) and 914 pediatric GIs (6.8%). Using physician workforce data published by professional societies, 88.9% (95% CI, 88.4%-89.4%) of board-certified adult GIs (12 558 of 14 126) and 66.7% (95% CI, 64.2%-69.2%) of board-certified pediatric GIs (914 of 1371) (*P* < .001) received some form of benefit from industry in 2016. Looking at total expenditures, the sum of listed payments was $67 144 862, with pediatric GIs receiving $1 811 825 (2.9%). As shown in Figure 1, the relative distribution of payments demonstrated that 89.9% of the payments (388 885 of 432 463) were related to food, with $8 344 513 (12.4% of expenses) being spent on 96.1% of all recipients (12 942 of 13 467) listed.

Consultation fees and compensation accounted for 18 179 (4.2%) of the payments but were responsible for 62.7% of the total expenditures and went to 2055 physicians (13.2%) identified by their unique identification number within the Open Payments database. Consultation fees accounted for 4302 of these payments and were made to 1242 GIs. Other categories contributed relatively little to the overall number of payments or expenditures, with 13.5% and 14.8% receiving support for travel and lodging or education, respectively (Figure 1), which accounted for less than 3% ($659 338 for 3409 payments) of the total expenditures. Conversely, royalties and licensing fees amounted to 11.0% of the annual expenses reported but were paid to only 22 individuals. Only 7 individuals received interest payments or other ownership benefits, which accounted for 1.7% of the annual expenditures listed.

### Personal Financial Rewards

**Figure 2.**
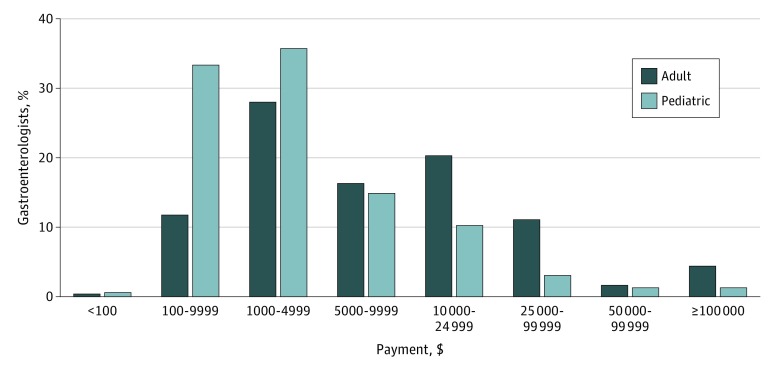
Relative Distribution of Direct Financial Rewards Paid by Industry Sources The sum of direct payments for consultative or other services to individual physicians is displayed in predefined brackets for adult and pediatric gastroenterologists. To facilitate comparisons, the number of recipients in each category is expressed as a percentage of the professional peer group.

Because consultation fees and other honoraria constitute a direct financial benefit and were responsible for nearly two-thirds of the total expenditures, we performed a more detailed analysis of these payments. We abstracted 18 179 payments (pediatric GI accounted for 3.3%) that fell into these categories and were linked to 2055 individuals (pediatric GI accounted for 8.2%), amounting to a total of $42 086 207. Relating these numbers to information about the physician workforce, 13.4% (95% CI, 12.8%-14.0%) of all adult GIs and 12.3% (95% CI, 10.2%-14.6%) of all pediatric GIs (*P* = .25) received personal financial rewards from pharmaceutical companies or device makers for consultative or other services in 2016. When we linked payments to individuals and categorized results based on distinct payment ranges, we noted that 819 adult GIs received more than $10,000, which amounts to 6.5% (95% CI, 6.1%-7.0%) compared with 26 pediatric GIs, which adds up to 2.8% (95% CI, 1.8%-4.1%), a significant difference (*P* < .001) (Figure 2). Considering the commonly used operational definition of relevant COI based on a threshold sum of $10 000, 43.4% of adult GIs (5439 of 12 533) and 15.5% of pediatric GIs (142 of 914) who received payments from pharmaceutical company or device manufactures, received amounts greater than $10 000.

**Figure 3.**
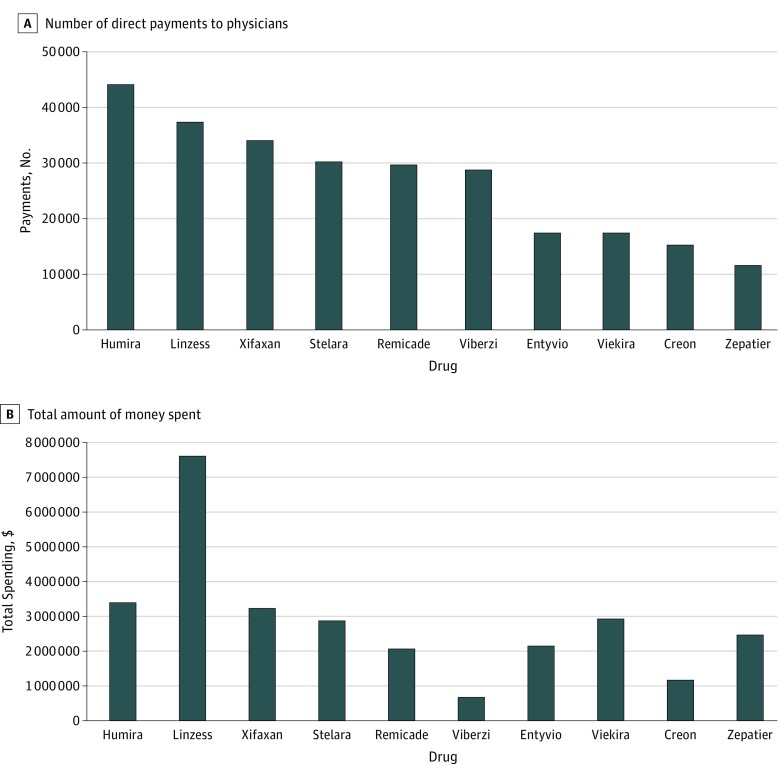
The 10 Most Common Drugs Linked to Payments, as Reported in the Open Payments Database The absolute number of direct payments to physicians (A) and the total amount of money spent in 2016 (B) are shown for the 10 products with the highest number of reports. Products are listed based on their trade names as recorded in the Open Payments database.

For adult GIs, 10 drugs accounted for 63.8% of the payments related to direct financial benefit (11 221 of 17 588) and 37.1% of all the expenditures for 2016 ($24 892 643 of $67 144 862). The list included 5 antivirals targeting hepatitis C and 3 biological agents used in the treatment of inflammatory bowel disease (Figure 3A and 3B). Examining annual sales and the relevance of different agents within the annual revenue stream, the results suggest that marketing strategies that involve physicians vary as rankings based on honoraria and fees do not correspond to sales volume or relevance in a company’s income (eFigure in the Supplement).

Using this list of products with the highest number of financial rewards for GIs, we specifically examined the relative role of consultation fees. As relevant contextual information, we determined whether a new or pending approval or newly approved indications potentially explained the need for consultative input. Six agents had been approved and marketed prior to 2016, with the remaining 4 drugs receiving this approval during 2016 (Table 1). The number of individual consultants varied from 0 to more than 200. Most of the consultation fees were paid for advice related to antiviral therapies used in the management of hepatitis C. The highest number of such consultations was linked to an agent approved in and marketed since 2014. As hepatology has only recently become a board-certified specialty, we only have estimates about the physician workforce in this field^57^ and cannot determine whether such payments went only to hepatologists. Keeping these caveats in mind, these results suggest that up to 20% of the nation’s hepatologists may have functioned as consultants for a single antiviral agent.

When we ranked physicians based on personal financial rewards obtained from industry sources, the top 25 GIs received a median (range) annual payment of $225 917 ($184 938-$299 063). Payments were primarily listed as compensation for services other than consultations, with a median (range) 90.2% (44.7%-100%) of the payments falling into this category. Most of the physicians in this group worked in academic institutions, with 8 (32.0%) in private practice. Reviewing the publication record for the years 2016 and 2017, physicians with the highest compensation rates had a median (range) of 3 (0-29) publications, with 5 physicians (20%) not listed as author on any article in the PubMed database for 2016 and 2017.

Considering the smaller number of pediatric GIs, we identified the 10 top earners, who received a median (range) of $34 097 ($18 450-$216 069), mostly paid as consultation fees (median [range], 85.6% [9.7%-100%]). Eight physicians worked in academic centers, while 2 were in private practice. Based on PubMed review, the median (range) number of listed publications was 4.5 (0-28), with 1 person having no publications in 2016 and 2017.

### Clinical Practice Guidelines

We identified 36 clinical practice guidelines that were published by the 4 professional organizations in 2016 or 2017 (Table 2). All but 1 article included a statement about COIs, with 4 articles providing a summary statement about the lack of any COI, 1 referring to detailed information obtained and retained at the organizational headquarters, and 1 declaring no competing interests. Twenty-nine publications included authors who were listed in the Open Payments database, with amounts exceeding $10 000 in 8 of 36 guidelines (22%). When we corrected for participation in multiple panels, a total of 99 individuals were identified, with 49 being listed in Open Payments as recipients of consultation fees or compensation. The median (range) payment was $0 ($0-$101 820), with 32 having received more than $10 000 and 7 panelists more than $100 000 in 2016. Nine of the top 10 award recipients were hepatologists who participated in panels on hepatitis C management (n = 5), diagnosis of acute liver failure (n = 2), abnormal liver enzymes (n = 1), or elastography (n = 1). All but 1 of these panelists received benefits linked to several direct-acting antiviral agents and rifaximin. One other panelist participated in the formulation of several guidelines related to colorectal cancer screening and surveillance and received payments linked to endoscopic devices.

**Table 2.** Clinical Practice Guidelines Published by the 4 Professional Organizations in 2016 or 2017, With Number of Authors and References

Source^a^	Organization	Topic	US Authors, No.^b^	Authors Who Received Direct Payments, No.^c^	Comment
Nguyen et al,^19^ 2016	AGA	IBD and pregnancy	1	1	
Kahi et al,^20^ 2016	AGA	CRC surveillance	10	3	Summary statement of no COI
Fallone et al,^21^ 2016	AGA	*Helicobacter pylori* management	0	0	
Wani et al,^22^ 2016	AGA	Barrett esophagus with low-grade dysplasia	2	1	No detailed COI statement
Regueiro et al,^23^ 2017	AGA	IBD therapy after surgery	6	3	
Enns et al,^24^ 2017	AGA	Use of capsule endoscopy	0	0	
Flamm et al,^25^ 2017	AGA	Therapy of liver failure	4	2	COI statement in central office
Herrine et al,^26^ 2017	AGA	Testing in acute liver disease	3	2	Summary statement of no COI
Freedberg et al,^27^ 2017	AGA	Proton pump inhibitor use	3	0	
Abu Dayyeh et al,^28^ 2017	AGA	Endoscopic therapy in bariatrics	3	2	
Robertson et al,^29^ 2017	AGA	FIT testing for CRC	10	4	
Jacobson et al,^30^ 2017	AGA	Care after HCV treatment	3	3	
Kanwal et al,^31^ 2017	AGA	HCV care team	8	6	
Singh et al,^32^ 2017	AGA	Elastography	4	3	
Rex et al,^33^ 2017	AGA	CRC screening	9	3	
Vande Casteele et al,^34^ 2017	AGA	IBD drug monitoring	4	2	Summary statement of no COI
Kahrilas et al,^35^ 2017	AGA	Per-oral endoscopic myotomy in achalasia	3	2	Summary statement of no COI
Shaheen et al,^37^ 2016	ACG	Barrett esophagus management	4	3	
Tran et al,^38^ 2016	ACG	Liver disease in pregnancy	3	3	
Kahi et al,^39^ 2016	ACG	CRC surveillance	9	3	
McClave et al,^40^ 2016	ACG	Nutritional support	4	1	
Strate and Gralnek,^41^ 2016	ACG	Lower gastrointestinal bleeding	2	1	
Riddle et al,^42^ 2016	ACG	Infectious diarrhea	3	1	
Kwo et al,^43^ 2017	ACG	Evaluation of abnormal liver enzymes	3	3	No competing interests
Robertson et al,^44^ 2017	ACG	FIT testing	10	4	
Chey et al,^45^ 2017	ACG	*Helicobacter pylori* therapy	3	2	
Farraye et al,^46^ 2017	ACG	Preventive care in IBD	4	4	
Durno et al,^47^ 2017	ACG	Biallelic mismatch mutation	9	3	
Moayyedi et al,^48^ 2017	ACG	Dyspepsia	3	3	
Rex et al,^49^ 2017	ACG	CRC screening	9	3	
Garcia-Tsao et al,^51^ 2017	AASLD	Portal hypertensive bleeding	2	1	
Jones et al,^53^ 2017	NASPGHAN	*Helicobacter pylori* treatment	2	0	
Vos et al,^54^ 2017	NASPGHAN	Nonalcoholic fatty liver disease	6	2	
Fawaz et al,^55^ 2017	NASPGHAN	Cholestasis in infants	6	2	
Krishnan et al,^56^ 2016	NASPGHAN	Esophageal atresia	2	0	

Abbreviations: AASLD, American Association for the Study of Liver Diseases; ACG, American College of Gastroenterology; AGA, American Gastroenterological Association; COI, conflict of interest; CRC, colorectal cancer; FIT, fecal immunochemical test; HCV, hepatitis virus C; IBD, inflammatory bowel disease; NASPGHAN, North American Society for Pediatric Gastroenterology, Hepatology and Nutrition.

^a^ Represents the reference to the guidelines that appear in the reference section of this article.

^b^ Shows number of US authors involved in formulating guidelines.

^c^ Shows number of authors listed as having received direct payments in the Open Payments database.

## Discussion

Our data demonstrate general acceptance of benefits from the pharmaceutical industry, as approximately 90% of GIs were listed in the Open Payments database. While most of these benefits were in the form of food and/or beverages and often limited in scale, direct payments of honoraria and consultation fees were financially more relevant, accounting for 4.2% of the transactions but 62.7% of the total industry expenditures listed in the database. We thus took a closer look at such direct monetary rewards and determined that 13.2% of GIs received direct payments from the pharmaceutical industry and 6.5% of adult GIs and 2.8% of pediatric GIs received an amount that exceeded $10 000. This pattern was noticeable when we examined contributors to clinical practice guidelines, with 32 having received more than $10 000. These results are higher than reported for the entire cohort of physicians practicing in the United States, as only about half received some industry support.^58^ In addition to the higher numbers of persons accepting rewards, the total amounts were higher than previously reported for other specialties.^10,58,59^ Thus, industry sources do not only target and reach more GIs, they also directly pay more of them, granting higher rewards for consultation or sponsored education.

We also identified differences between physicians practicing within the specialty of gastroenterology, but focusing on different patient populations, namely pediatric and adult patients. Differences in age-specific disease prevalence likely explain some of these findings. For example, antiviral agents targeting hepatitis C were among the agents linked to the highest expenditures related to physician payments but play a marginal role in the treatment of pediatric patients. However, differences persisted when we looked at biological agents used in the management of inflammatory bowel disease. The data available in the repository do not permit clear conclusions about distinct targeting strategies that go beyond disease prevalence or limited drug approval.

The common practice of accepting benefits from industry sources stands in apparent contrast with the increasing awareness of possible COIs and implementation of institutional policies governing such potential conflicts. However, these findings confirm polls that show ongoing approval of sponsored meals and compensation for talks or consultative interactions between physician and industry.^60,61^ When faced with the likely biasing impact of financially lucrative interactions with industry, surveyed physicians recognized the potential purpose and result of such rewards, but typically saw themselves as less vulnerable to such influence, despite evidence supporting the opposite.^61,62^ This commonly held perception is not consistent with systematic studies. Even only relatively small benefits affect behavior, as shown by correlations between meal rewards and prescription patterns for specific products.^63^ Direct payments in the form of compensation or consultation fees showed a more notable association with prescribing patterns than did simply receiving a free meal.^64^ A more detailed analysis of consultation fees related to 1 of 2 specific agents found a strong association with the number of prescriptions for the very agent linked to payments the prescriber had received.^65^ These recent studies are consistent with reviews that highlighted the influence of industry strategies ranging from sales personnel to grant funding, all of which affect physician behavior.^66,67^ Thus, even small contributions, such as sponsored meals, matter. We can only speculate about the effects of more significant support, such as honoraria and fees. Beyond a direct influence on recipient behavior, sponsored talks or support of known experts will likely go beyond the direct beneficiary, as known experts typically express their opinions in talks and publications.

Our data clearly show the relative importance of direct financial rewards for physicians, which is consistent with findings in other specialties,^65,68,69^ and accounted for nearly two-thirds of all expenditures reported to the Open Payment repository. Most of these payments were listed as compensation for talks and presentations. While such activities can indeed support the education of physicians and other audiences, company-organized speakers’ bureaus have come under criticism, as they often promote specific products, at least in part based on the speaker’s prestige or rhetorical ability, rather than evidence or suitability.^70^

Approximately one-quarter of the payments and expenditures were labeled consultation fees, thus falling into the very category that prompted investigations by the Department of Justice^7^ and ultimately led to the creation of the Open Payments database. The database does not include information about the frequency, duration, or content of consultative interactions. We can thus not assess the value of these interactions beyond the literal value in dollars. However, it is difficult to understand why companies extensively consult about drugs or devices that have been approved and marketed for many years. An examination of annual sales and their relative contribution to company revenues suggests that different factors contribute, as the ranking based on expenses does not seem to correlate with sales or relative value of a product for the company. While we can only speculate, strategic arguments from competitive pressures to the product spectrum or introduction of a new product will all contribute to decisions about marketing. The number of consultants for some agents and at times repeated rewards within a single year raise questions about what information is solicited, how this information is processed, and how it provides new insight, especially as some individuals consulted have no apparent record of unique expertise, as operationally defined by publications on topics related to the product of interest.

Perhaps related to the known or perceived influence on physician behavior and advice,^71^ knowing about industry rewards erodes patient trust in physicians^72^ and affects patients’ views about the conduct and results of clinical studies.^73^ It also leads to questions about the formulation of clinical guidelines or standards, as experts often receive benefits from industry and are not immune to the biases discussed in this article. Recent studies showed a potential COI for more than 80% of the contributors to guidelines in oncology or dermatology and identified at times incomplete information about relevant rewards or funding.^12,74^ Within the last 2 years, guidelines published by professional organizations representing the field of gastroenterology consistently declared potential COIs with sufficiently detailed information in most instances. However, about half of the panelists had received direct payments from industry sources and almost one-third had accepted more than $10 000 in 2016. We focused on direct personal gain, rather than grants or research support; grants and research support may also influence physician behavior but can potentially benefit patients. In addition, we only examined the details of industry funding and did not correlate it with differences in prescriptions or other forms of medical practice.

### Limitations

Our data are limited to numbers and categories, but not the exact nature or even content of the interactions for which physicians were paid. The wide range of payments falling under similar categories suggests not only a different value, as perceived by the awarding company, but likely also a different nature of such interactions. Compensations for presentations or other activities similarly covered a range that differed by a factor of 10 or more. As regulations do not require more detailed information, surrogate markers, such as publication records or academic affiliation, could shed light on some of the underlying reasons for these differences. We only examined such data for the small subgroup of top earners and further limited our approach to the most recent 2 years.

We focused on guidelines to assess whether there was a potential COI due to payments that panelists received from industry. We restricted our analysis of payments and participation in guideline panels to a relatively short period that may not sufficiently capture potential influences. The topics covered within the time frame of our analysis were all authored by multiple individuals, generally based on systematic analyses, and often relied on meta-analytic techniques, which limited potential bias. A limitation that originates from the Open Payments database itself is the lack of information about non-US physicians and some degree of incomplete and inaccurate reporting of payments. It would be interesting to focus on reviews, editorials, or opinion pieces, which have a more limited number of contributors, are not based on a rigorous methodology, and may thus be more skewed by the effects of paid consulting or other services.^75^

## Conclusions

Our study highlights the common involvement of GIs in activities that come with financial gain, which may indirectly or directly influence advice given to patients or colleagues and that, thus, constitute potential COIs. Mandated declarations and the creation of the Open Payments database provide more transparency for those searching for information about potential COIs. However, our findings argue against the underlying assumption that transparency by itself or the resulting self-regulation by the parties involved significantly reduced or even eliminated such conflicts, as the number of GIs accepting any form of benefit or receiving honoraria or fees is higher than reported for other groups of physicians. Considering the importance for innovation and the potential benefit for patients, we should not simply curb the relationship between industry and physicians. Instead, we could strengthen mechanisms that monitor and report on possible COIs. One important component should involve the presentation of such information, which could move from difficult-to-decipher fine print to graphically displayed, easily understandable data that place financial or other rewards in a relevant context, such as peer-group medians. Future research should focus on the question of how improved transparency of potential COI affects authors and presenters or their respective target audiences.

## Notes

Supplement. **eFigure.** Annual Sales
