# Supplementary material for: Assessment of Pharmaceutical Company and Device Manufacturer Payments to Gastroenterologists and Their Participation in Clinical Practice Guideline Panels
Source: JAMA Netw Open. 2018 Dec 28;1(8):e186343. doi: 10.1001/jamanetworkopen.2018.6343 (PMC6324539; doi:10.1001/jamanetworkopen.2018.6343)
Supplement: Supplement. — eFigure. Annual Sales [file jamanetwopen-1-e186343-s001.pdf]

Supplementary Online Content

Nusrat S, Syed T, Nusrat S, Chen S, Chen W-J, Bielefeldt K. Assessment of pharmaceutical company and device manufacturer payments to gastroenterologists and their participation in clinical practice guideline panels. *JAMA Netw Open*. 2018;1(8):e186343. doi:10.1001/jamanetworkopen.2018.6343

eFigure. Annual Sales

This supplementary material has been provided by the authors to give readers additional information about their work.

© 2018 Nusrat S et al. *JAMA Network Open.*

Annual sales within the United States (A) and their relevance in reported revenues (B) are shown for the ten drugs with the highest cumulative payments for fees and honoraria to gastroenterologists. The drugs are ranked based on annual payments as listed in Table 1.

eFigure. Annual Sales

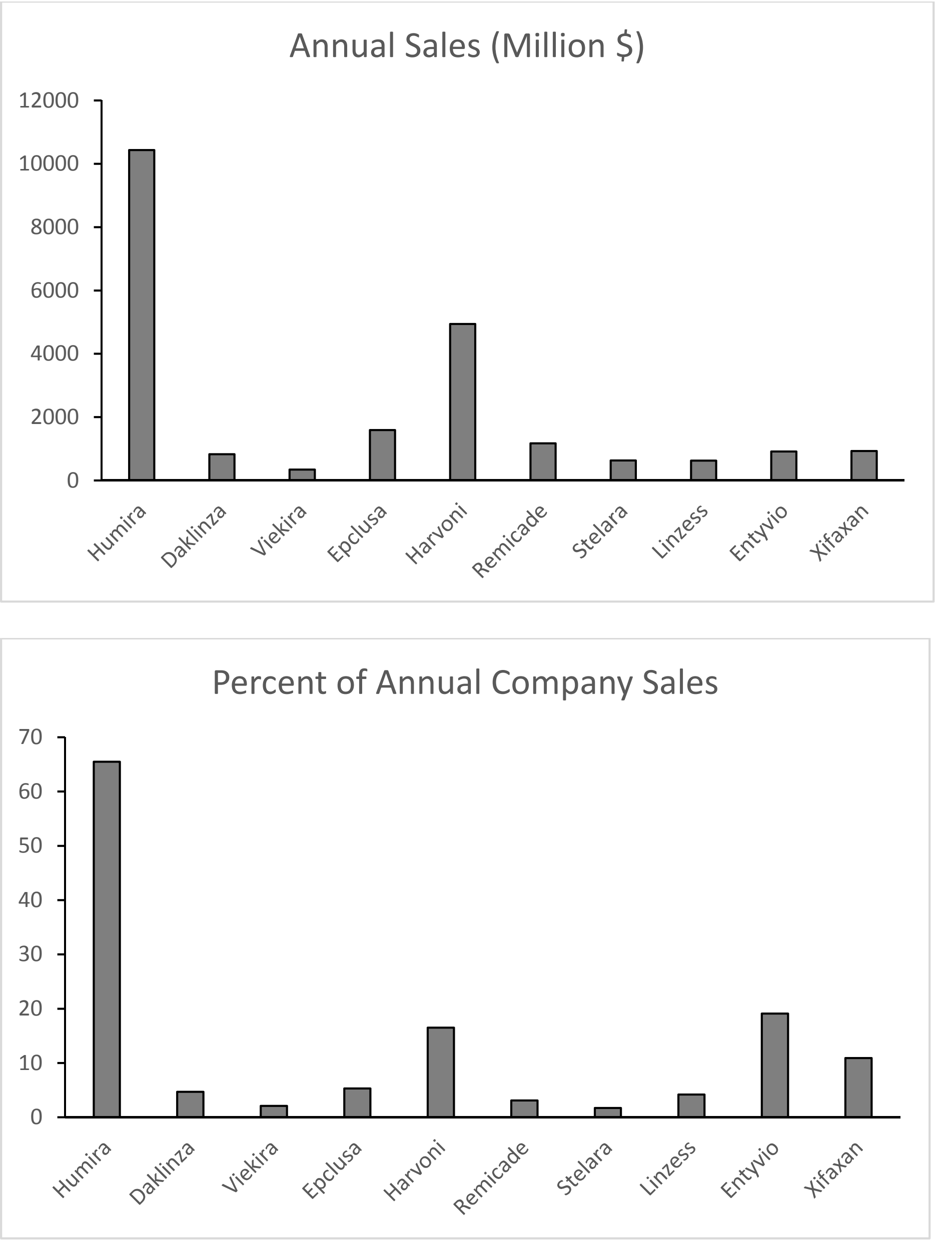

© 2018 Nusrat S et al. *JAMA Network Open.*

Data were extracted from publicly available investor reports accessed on 10/26/2018 listed below.

https://news.bms.com/press-release/financial-news/bristol-myers-squibb-reports-fourth-quarter-and-full-year-2016-financia

https://www.gilead.com/news/press-releases/2017/2/gilead-sciences-announces-fourth-quarter-and-full-year-2016-financial-results

http://www.investor.jnj.com/secfiling.cfm?filingID=200406-17-6&CIK=200406

http://investor.ironwoodpharma.com/news-releases/news-release-details/ironwood-pharmaceuticals-provides-fourth-quarter-and-full-year-0

https://www.allergan.com/investors/news/thomson-reuters/allergan-reports-strong-2016-finish-with-7-increas

http://ir.valeant.com/~/media/Files/V/Valeant-IR/reports-and-presentations/q4-fy2016-vrx-02282017-v1.pdf

http://ir.valeant.com/news-releases/2017/02-28-2017-120547378

https://www.takeda.com/siteassets/system/investors/report/quarterlyannouncements/fy2016/fy-2016-q4-announcements-released-on-may-10-2017/qr2016_q4_d1_en.pdf

© 2018 Nusrat S et al. *JAMA Network Open.*
